# Sequential administration of anti-PD-1 and anti-Tim-3 combined with an SA-GM-CSF-anchored vaccine overcomes adaptive immune resistance to reject established bladder cancer

**DOI:** 10.7150/jca.44769

**Published:** 2021-02-02

**Authors:** Xinji Zhang, Guang Liu, Xianghua Shi, Xiaojun Shi, Jinlong Li, Lijun Mo, Jimin Gao, Zhaolin Long, Wanlong Tan

**Affiliations:** 1 Department of Urology, Nanfang Hospital, Southern Medical University, Guangzhou, 510515, China.; 2 Department of Urology, Shunde Hospital, Southern Medical University (The First People's Hospital of Shunde District, Foshan), Foshan, 528300, China.; 3 Department of Urology, The First People's Hospital of Foshan, Guangdong,528000, China.; 4 Institute of Biotherapy, School of Laboratory Medicine and Biotechnology, Southern Medical University, Guangzhou, 510515, China.; 5 Zhejiang Provincial Key Laboratory of Medical Genetics, School of Life Sciences, Wenzhou Medical University, Wenzhou, 325035, China.

**Keywords:** program death receptor 1, T cell immunoglobulin and mucin domain-containing protein-3, bladder cancer, vaccine, immunotherapy

## Abstract

Program death receptor-1 (PD-1) and T-cell immunoglobulin and mucin domain-containing protein-3 (Tim-3) play an important role in tumor immune evasion. PD-1 blockade could produce an effective anti-tumor effect but the response rate was low due to lacking of tumor infiltrating lymphocytes (TILs) and existing of other negative regulatory pathways. Streptavidin(SA)-GM-CSF surface-anchored tumor cells vaccine could induce specific anti-tumor immune response. However, this vaccine failed to induce regression of established tumor because it also up-regulated PD-1 expression on tumor cells dependent on IFNγ and up-regulated PD-1/Tim-3 expression on CD8^+^ TILs. Subsets of CD8^+^ TILs assay showed that PD-1 expression was closely associated with CD8^+^ TILs exhaustion, and Tim-3 expression was closely correlated with secretion function but not proliferation of CD8^+^ TILs. Sequential administration of anti-PD-1 and anti-Tim-3 could further improve the efficacy of SA-GM-CSF-anchored vaccine therapy, and tumor regression was noted in over 50%. This triple therapy improves the specific cytotoxic activity and decreased the apoptosis of CD8^+^ TILs. These findings indicated that this triple therapy could induce a more robust anti-tumor immune response.

## Introduction

Bladder cancer is a common malignancy of the urinary tract and is characterized by high rates of recurrence and progression 1. Routine chemotherapy cannot effectively improve the outcome for metastatic urothelial cancer 2. With the development and innovation of immunotherapy, therapeutic vaccines have been demonstrated to be effective in enhancing the endogenous tumor-reactive immune response 3. In our previous studies, based on the unique property of streptavidin (SA) to bind rapidly and almost irreversibly to any biotin-linked molecule and the outstanding ability of biotin to be easily incorporated into proteins on the cell surface 4, we developed a protein anchor platform to immobilize SA-tagged bioactive molecules on the surface of biotinylated MB49 bladder cancer cells. We also confirmed that cytokine-modified MB49 bladder cancer cell vaccines could effectively induce a specific antitumor immunity that inhibited the growth of bladder cancer in an MB49 mouse model 5. However, we also found that the majority of the vaccine-treated tumors slowly regrew over long-term observation. A possible reason may be that immune resistance exists in the tumor microenvironment (TME).

As reported by several studies, program death receptor-1 (PD-1)/program death ligand 1 (PD-L1) signaling constitutes a major tolerance mechanism 6. The PD-1/PD-L1 interaction could result in exhaustion of antitumor effector T cells. Exhausted T cells are routinely characterized by sustained expression of the inhibitory molecule of PD-1 and failure to proliferate and exert effector functions in response to antigen stimulation 7. The clinical efficacy of blocking PD-1/PD-L1 was recently shown in several advanced malignancies 8,9. However, several clinical studies of bladder cancer have demonstrated that targeting the PD-1 pathway does not always result in reversal of T cell exhaustion, and the response rate was unsatisfactory 10. Possible reasons for this result were associated with a lack of initial tumor-infiltrating lymphocytes (TILs) and the involvement of other negative regulatory pathways in exhaustion of effector T cells.

T-cell immunoglobulin and mucin domain protein-3 (Tim-3) is one of the members of the Tim family and is selectively expressed on Th1/Tc1 T cells 11. The role of Tim-3 in the immune regulation of tumors has been confirmed by many studies. Interaction between Tim-3 and its ligand galectin-9 can trigger cell death in Th1 cells and induce peripheral tolerance 12. Recently, several studies have found that Tim-3 is overexpressed in several types of human tumors, such as prostate 13, gastric 14 and bladder cancer 15, and its overexpression is negatively correlated with the prognosis of these cancers. Concurrent Tim-3 blockade could enhance the effect of PD-1 blockade in restoring TIL functionality 16.

Until now, whether PD-1 and Tim-3 are expressed by SA-granulocyte-macrophage colony-stimulating factor (SA-GM-CSF)-anchored vaccine-induced tumor antigen-specific CD8^+^ TILs and whether they play a role in regulating the proliferation and cytokine secretion functions of the CD8^+^ TILs in bladder cancer remain unknown. Based on the roles of the SA-GM-CSF-anchored vaccine in antigen-specific CD8^+^ T cell activation 5 and the fact that activated T cells express a variety of inhibitory receptors, we speculated that PD-1 blockade may synergize with the SA-GM-CSF-anchored vaccine to enhance the tumor antigen-specific CD8^+^ T cell response, and sequential administration of PD-1 and Tim-3 blockades in SA-GM-CSF-anchored vaccine therapy may hold greater potential for eliciting an immune response to eliminate bladder cancer. In this study, we sought to detect the expression of PD-1 on CD8^+^ TILs and PD-L1 in the TME during SA-GM-CSF-anchored vaccine treatment and explored the functional exhaustion status of CD8^+^ TILs based on PD-1 and Tim-3 expression. Furthermore, we evaluated the efficacy of sequential administration of PD-1 and Tim-3 blockades in SA-GM-CSF-anchored vaccine therapy (triple therapy) in an MB49 model.

## Materials and Methods

### Animals and cells

C57BL/6 mice (6-8 weeks) were purchased from the experimental animal center of Southern Medical University (Guangzhou, China). The MB49 cell line was a carcinogen-induced transitional cell carcinoma derived from C57BL/6 male mice. The MB49 cells were cultured in RPMI 1640 containing 10% fetal bovine serum and 1% penicillin/streptomycin in a 5% CO_2_ humidified incubator. MycoplasmaOUT (Genloci, Nanjing, CHN) was used to protect the cells against mycoplasma contamination. The MB49 cells were authenticated and tested for mycoplasma contamination every 6-12 months. All animal studies were performed in accordance with the university guidelines for experimental animals (ethical number: L2016045). The SA-GM-CSF fusion protein and SA-green fluorescent protein (SA-GFP) were prepared by our laboratory.

### Vaccine preparation and bioactivity assay

According to our previous studies 5, MB49 cells in the exponential growth stage were fixed in 30% ethanol (volume/volume) for 30 minutes and then incubated with 10 mM EZ-Link® Sulfo-NHS-LC-Biotin (Pierce Biotechnology, Rockford, USA) for 1 hour at room temperature and washed 3 times with PBS containing 100 mM glycine. Then, the biotinylated MB49 cells were incubated with SA-GM-CSF at 100 ng/10^6^ cells for 1 hour and washed 3 times with PBS. The percentage of SA-GM-CSF expression on the surface of MB49 cells was assayed by flow cytometry. The bioactivity of SA-GM-CSF immobilized on the surface of MB49 cells was assessed by bone marrow cell proliferation, and SA-GFP was used as a negative control.

### Establishment of the tumor model and treatment assay

**Figure 1:**
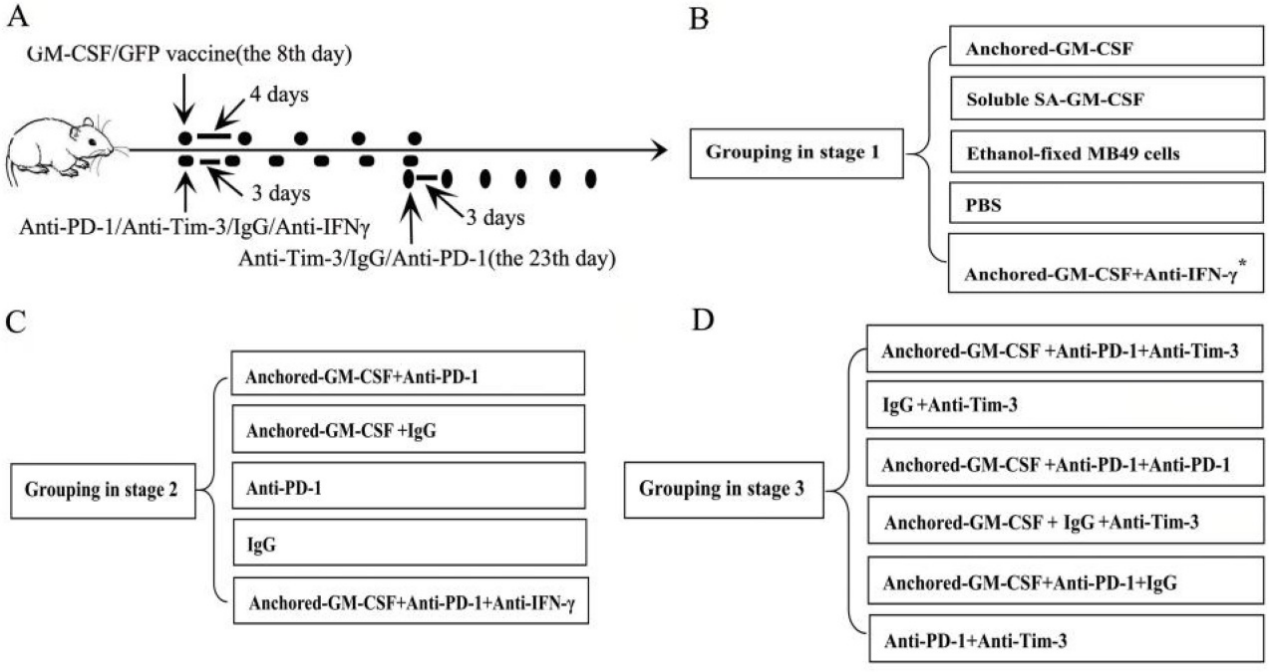
** Detailed information of grouping and treatment.** (A) Detailed diagram of tumor therapy. (B, C, D) The grouping at different stages. The dosage and frequency of treatment: SA-GM-CSF-anchored vaccine: 1×10^6^ cells/100 µl for each dose, once every 4 days for 5 doses. Soluble SA-GM-CSF: 100 ng/100 µl for each dose, once every 4 days for 5 doses. Ethanol-fixed MB49 cells: 1×10^6^ cells/100 µl for each dose, once every 4 days for 5 doses. PBS: 100 µl for each dose, once every 4 days for 5 doses. Anti-IFNγ antibody: 100 µg for each dose, once every 3 days for 6 doses. Anti-PD-1 antibody (eBioscience): 100 µg for each dose, once every 3 days for 6 doses. IgG antibody (eBioscience): 100 µg for each dose, once every 3 days for 6 doses. Anti-Tim-3 antibody (Invitrogen): 100 µg for each dose, once every 3 days for 6 doses. The routes of administration for the SA-GM-CSF-anchored vaccine, soluble SA-GM-CSF, ethanol-fixed MB49 cells, and PBS: intracutaneous injection; for the anti-IFNγ antibody, anti-PD-1 antibody, IgG antibody, and anti-Tim-3 antibody: intraperitoneal injection. ^*^: when analyzing the expression of PD-L1 in the TME, anti-IFNγ antibody treatment was added.

A total of 1×10^6^ MB49 cells suspended in 100 µl of PBS were injected subcutaneously into the hind leg of C57BL/6 mice to establish subcutaneous models. This part of the study was divided into three stages. The first stage was to evaluate the efficacy of the SA-GM-CSF-anchored vaccine. In this stage, when analyzing the expression of PD-L1 in the TME, an anti-IFNγ antibody (XMG1.2, Biolegend, San Diego, USA) was added. The second stage was to evaluate the efficacy of PD-1 blockade in treatment with the SA-GM-CSF-anchored vaccine. The third stage was to evaluate the efficacy of sequential administration of anti-PD-1 (J43, eBioscience, San Diego, USA) and anti-Tim-3 (F38-2E2, eBioscience, San Diego, USA) in treatment with the SA-GM-CSF-anchored vaccine. The treatment was started once palpable tumors developed (8 days after MB49 cells injection). The detailed information of grouping and treatment is shown in Figure 1.

Subcutaneous tumor growth was measured every three days, and the volume was calculated by the formula [π/6(w1×w2×w2)], where w1 represents the largest and w2 represents the smallest tumor diameter 5. Mice were sacrificed when the tumor size was larger than 20 mm in its greatest dimension.

### Maturation analysis of dendritic cells (DCs) after SA-GM-CSF-anchored vaccine treatment

After SA-GM-CSF surface-anchored vaccine treatment was finished, splenocytes were isolated and red blood cells were lysed. The splenocytes were stained with FITC-labeled anti-mCD11c (N418, eBioscience) and PE-labeled anti-mCD80 antibodies (16-10A1, eBioscience). The detailed step could be found in our previous research 17.

### Tumor-infiltrating lymphocyte isolation

Fresh tumors in each group were whittled into small pieces and then transferred to 70-μm cell strainers (BD, New Jersey, USA) and separated using the plunger of a 5-ml syringe. The cells passing through the cell strainer were collected and subjected to Ficoll-Hypaque gradient centrifugation. After centrifugation, TILs were recovered.

### Tim-3 expression analysis and CD8^+^ T cell subset sorting

The TILs were purified by using CD8 Microbeads (Miltenyi Biotec, Auburn, CA). For the analysis of Tim-3 expression, CD8^+^ TILs were stained with a PE-labeled anti-Tim-3 antibody (B8.2C12, Biolegend) and assessed by flow cytometry. For CD8^+^ TIL subset sorting, after staining with APC-labeled anti-PD-1 (29F.1A12, Biolegend) and PE-labeled anti-Tim-3 antibodies (B8.2C12, Biolegend), PD-1^+^Tim-3^+^, PD-1^+^Tim-3^-^, PD-1^-^Tim-3^+^ and PD-1^-^Tim-3^-^ CD8^+^ T cells were sorted using a Beckman Coulter MoFlo Astrios instrument.

### Proliferation and secretion functional analysis

For the analysis of proliferation function, CD8^+^ T cell subsets were collected and stained with an antibody against Ki67 (11F6, Biolegend) after permeabilization. For the analysis of secretion function, CD8^+^ T cell subsets were independently cocultured with anti-CD3-biotin (17A2, Biolegend) and anti-CD28-biotin (37.51, Biolegend). After permeabilization, these CD8^+^ T cells were independently stained with antibodies against IFNγ (XMG1.2, Biolegend) and TNFα (MP6-XT22, Biolegend). Then, cells were analyzed by flow cytometry.

### Cytotoxicity assay

Splenocytes were isolated from each group and stimulated by MB49 cells in the presence of interleukin-2 (IL-2) for 5 days as effector cells. MB49 cells served as target cells seeded in 96-well plates. A different number of effector cells were added to the target cells at the desired effector-to-target ratios. The supernatant was collected after 4 hours of incubation. Lactate dehydrogenase (LDH) activity was measured by the CytoTox 96® nonradioactive cytotoxicity assay (BD, New Jersey, USA). The experiment was repeated with RM-1 prostate cancer cells (for specificity analysis).

### Immunohistochemistry (IHC)

After the treatment was finished, subcutaneous tumor tissues were collected from each group and fixed with 4% paraformaldehyde and then embedded in paraffin. Paraffin sections (4-5 μm) were deparaffinized, rehydrated and treated with hydrogen peroxide, followed by antigen retrieval. After blocking for 10 minutes, the sections were incubated with anti-mCD8 (4SM16, eBioscience) according to instructions of the Rabbit-specific HRP/DAB (ABC) Detection IHC Kit (Abcam, Cambridge, UK) and restained with hematoxylin and eosin.

### Immunofluorescence staining

After antigen retrieval for 10 minutes as in the above procedure, the sections were incubated with rabbit anti-mPD-L1 (MIH6, Abcam) for 60 minutes. Then, an Alexa Fluor 647-labeled goat antirabbit secondary antibody (Abcam) was used. Slides were washed three times and mounted with a Vectashield DAPI containing kit (Vectorlabs, Shenzhen, CHN). The expression of PD-L1 in the TME was observed through a fluorescence microscope (Nikon, JPN).

### Enzyme-linked immunosorbent assay (ELISA)

After the treatment was finished, 100 μl peripheral blood was collected from each group and congealed at room temperature for 20 minutes. The supernatants were harvested by centrifugation at 3000 r.p.m. for 5 minutes. The concentrations of IL-6 and progranulin (PGRN) were measured by ELISAs (Abcam).

### Statistical analysis

All experimental group values were analyzed from representative experiments. The differences in tumor volume between groups were compared using a repeated measures design. Statistical analyses of flow cytometry, ELISA, IHC and immunofluorescence data were performed by one-way ANOVA. Statistical analysis was performed using SPSS (version 19.0). *P*<0.05 was considered statistically significant.

## Results

### SA-GM-CSF-anchored vaccine increased cytotoxic T lymphocyte (CTL) response but failed to induce regression of established tumors

**Figure 2:**
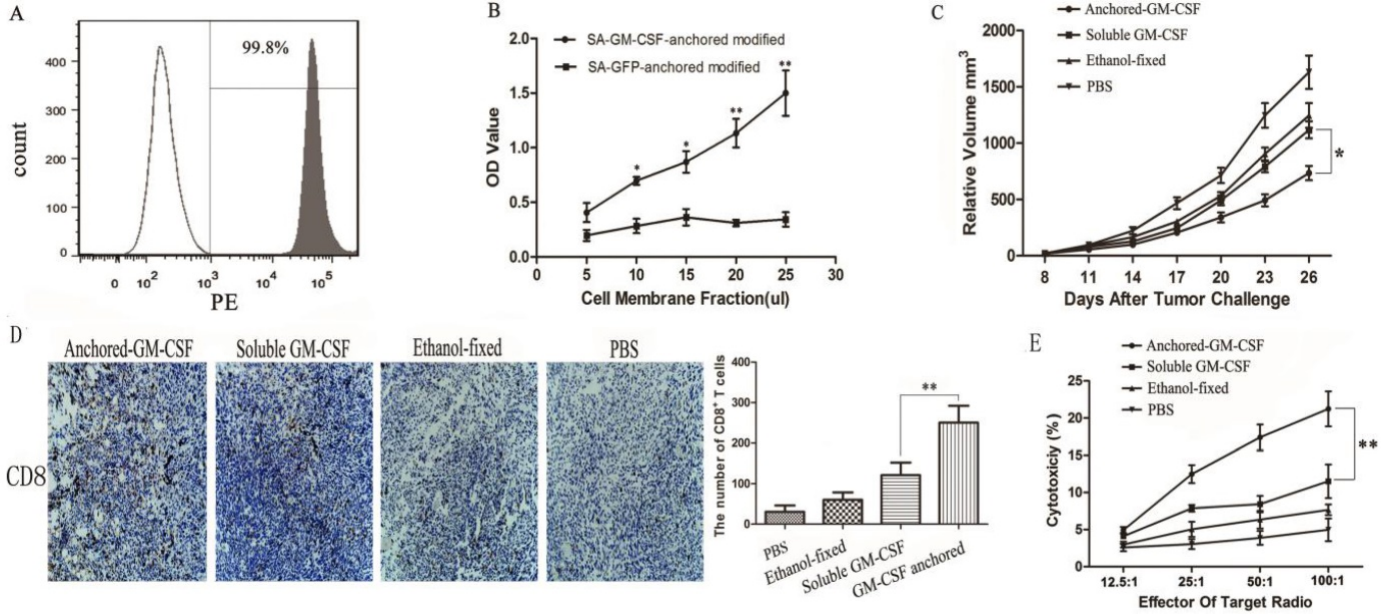
** Anchored-GM-CSF vaccine induced an antitumor response.** (A) The presence of SA-GM-CSF on the surface of MB49 cells was assayed by flow cytometry. Unbiotinylated MB49 cells served as a negative control. (B) The proliferative activity of membrane-conjugated GM-CSF on bone marrow cells was assessed, with SA-GFP as a negative control (*P<0.05, **P<0.01). OD: optical density. (C) All mice were injected subcutaneously with 1×10^6^ MB49 cells, and the tumor volume was recorded. The SA-GM-CSF-anchored vaccine effectively reduced the tumor growth compared with the other control groups but still failed to induce regression of established tumors (*P<0.05). (D) After SA-GM-CSF-anchored vaccine treatment, CD8^+^ T cell expression in the TME from each group was analyzed by IHC. The sections are presented as the percentages of the total vital tumor tissue (200×; **P<0.01). (E) After SA-GM-CSF-anchored vaccine treatment, splenocytes were isolated and stimulated by IL-2. MB49 cells served as target cells. Supernatants were collected for a nonradioactive cytotoxicity assay (**P<0.01). All the experiments were replicated 3 times.

Flow cytometry revealed that SA-GM-CSF could be efficiently anchored on the surface of MB49 cells (Figure 2A) and retain bioactivity (Figure 2B).

We evaluated the antitumor effect of the SA-GM-CSF-anchored vaccine in established subcutaneous models. The results showed that the SA-GM-CSF-anchored vaccine effectively reduced tumor growth but still failed to induce regression of established tumors (Figure 2C).

In our previous study, we confirmed that the SA-GM-CSF-anchored vaccine significantly increased the mature DC population 17. CD8^+^ T cells in the TME were detected by IHC. The number of CD8^+^ T cells in the SA-GM-CSF-anchored vaccine group was obviously increased compared to that in the other groups (Figure 2D).

To further investigate the antitumor immunity induced by SA-GM-CSF-anchored vaccine therapy, LDH activity was used to assess the cytotoxicity of CTLs. The results showed that the SA-GM-CSF-anchored vaccine effectively improved the cytotoxic activity (Figure 2E).

### SA-GM-CSF-anchored vaccine upregulated PD-1 expression on CD8^+^ TILs and PD-L1 expression in the TME

**Figure 3:**
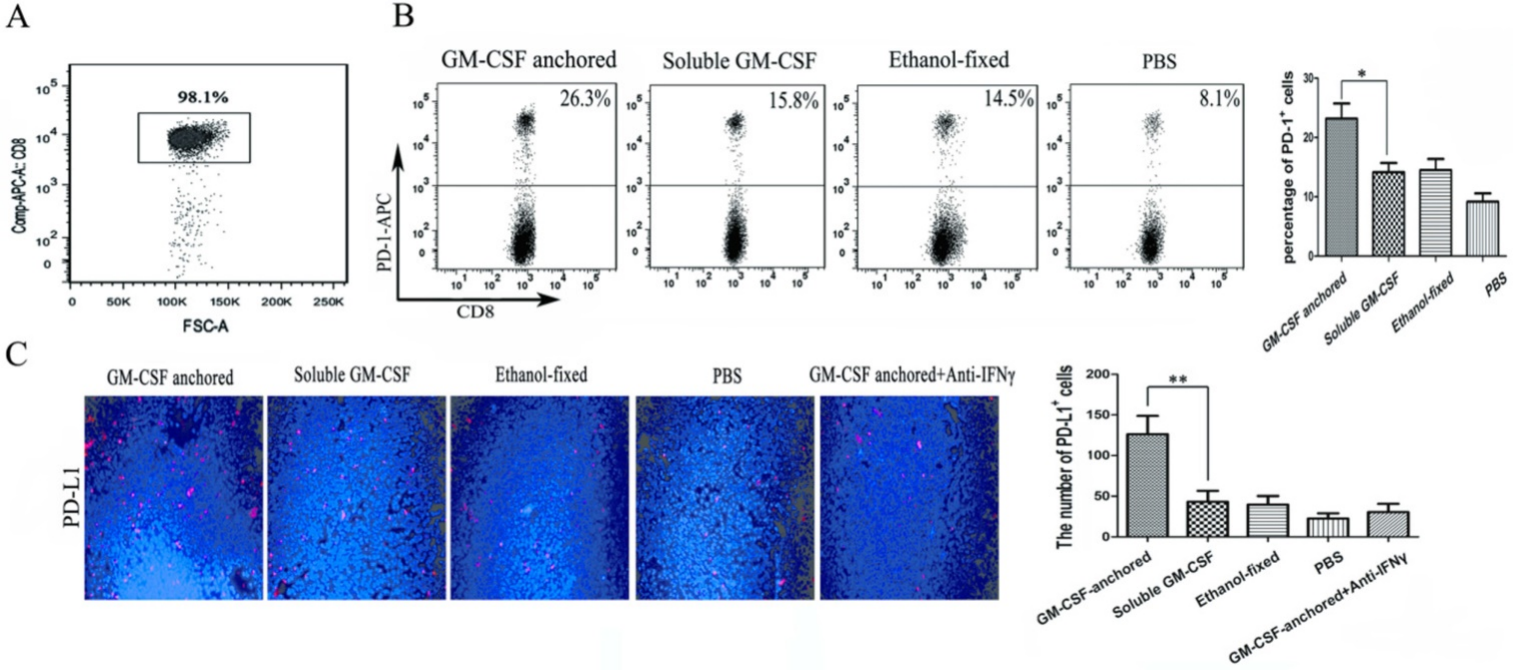
** Expression of PD-1 on CD8^+^ TILs and PD-L1 expression in the TME after Anchored-GM-CSF vaccine treatment.** (A) After treatment, TILs were isolated from tumor tissues of each group and purified by CD8a (Ly-2) MicroBeads. The sorting rate of CD8**^+^**TILs was detected by flow cytometry. (B) The CD8**^+^**TILs were stained with antibodies against PD-1, and PD-1 expression was assessed by flow cytometry (**P<0.01). (C) Tumor tissues from each group were harvested and stained with an anti-mPD-L1 antibody. Then, a goat antirabbit secondary antibody was used to assess PD-L1-positive cells. Blinded quantitation was performed in 10 different fields at 40× magnification (**P<0.01). All the experiments were replicated 3 times.

Immune checkpoint receptors expressed by T cells can negatively control their activation, expansion and effector functions through inhibitory signals generated by interacting with their cognate ligands. Many researchers hold the view that high expression of immune checkpoint receptors is a feature of T cell exhaustion 18. In this study, to evaluate the expression of PD-1 on CD8^+^ TILs, CD8^+^ T cells were first isolated from tumor tissues, and the isolation rate was 98.1% (Figure 3A). Then, the frequency of PD-1 expression on CD8^+^ TILs was assessed by flow cytometry, and the results showed that the SA-GM-CSF-anchored vaccine significantly upregulated the expression of PD-1 on CD8^+^ TILs (Figure 3B).

In our previous study, we confirmed that the SA-GM-CSF-anchored vaccine significantly increased the secretion of IFNγ in CD8^+^ T cells and serum 19. To confirm the adaptive immune resistance in SA-GM-CSF-anchored vaccine treatment, in this study, we performed an *in vivo* experiment and found that the SA-GM-CSF-anchored vaccine significantly upregulated the expression of PD-L1 in the TME by staining the tumor tissue, but this increase in PD-L1 expression was significantly diminished when treated with an IFNγ-neutralizing antibody (Figure 3C).

### PD-1 blockade reversed the associated adaptive immune resistance of the SA-GM-CSF-anchored vaccine in the form of a low response rate due to upregulated Tim-3 expression

**Figure 4:**
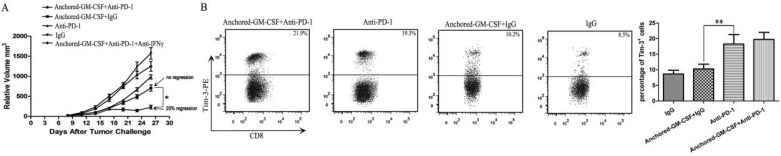
** PD-1 blockade further reduced the tumor growth in the form of a low response rate due to upregulated Tim-3 expression.** (A) The combination therapy of the Anchored-GM-CSF vaccine and PD-1 blockade further reduced the tumor growth compared to control groups (*P<0.05), but tumor regression occurred in only a few mice. (B) After the treatment was finished, CD8^+^ T cells were isolated from tumor tissues of each group and stained with a PE-labeled anti-Tim-3 antibody. Then, Tim-3^+^ expression on CD8^+^ TILs was assessed by flow cytometry (*P<0.05). All the experiments were replicated 3 times.

In this study, an anti-PD-1 antibody was used to reverse the adaptive immune resistance in SA-GM-CSF-anchored vaccine treatment. The results revealed that this combination therapy had synergistic effects and further reduced the tumor growth when compared to the SA-GM-CSF-anchored vaccine or anti-PD-1 antibody alone, and even the regression of established tumors was observed (Figure 4A). However, tumor regression occurred in only a few mice (20% regression), and this increased antitumor effect was significantly diminished when the mice were treated with an IFNγ-neutralizing antibody. The majority of the mice exhibited tumor progression eventually. This result was consistent with a recent clinical study of bladder cancer, which found that targeting the PD-1 pathway did not always result in the reversal of T cell exhaustion 10. Several studies have demonstrated that PD-1 blockade could upregulate Tim-3 expression in head and neck cancer 20 and lung cancer 21. In addition, the level of upregulated Tim-3 expression was closely related to the function of CD8^+^ T cells 18. To explore the reason for the low response rate to the combination therapy with PD-1 blockade and the SA-GM-CSF-anchored vaccine, we focused on the checkpoint expression of Tim-3 on CD8^+^ TILs when the treatment was finished. CD8^+^ T cells were isolated from tumor tissues, and the frequency of Tim-3^+^ CD8^+^ TILs was assessed by flow cytometry. The results showed that Tim-3 expression on CD8^+^ TILs was significantly increased in the anti-PD-1+SA-GM-CSF-anchored group and the anti-PD-1 group. No difference in Tim-3 expression on CD8^+^ TILs was observed between the SA-GM-CSF-anchored+IgG and IgG groups (Figure 4B).

### PD-1 expression alone was associated with CD8^+^ TIL exhaustion, while Tim-3 expression alone was associated with low secretory function of CD8^+^ TILs

**Figure 5:**
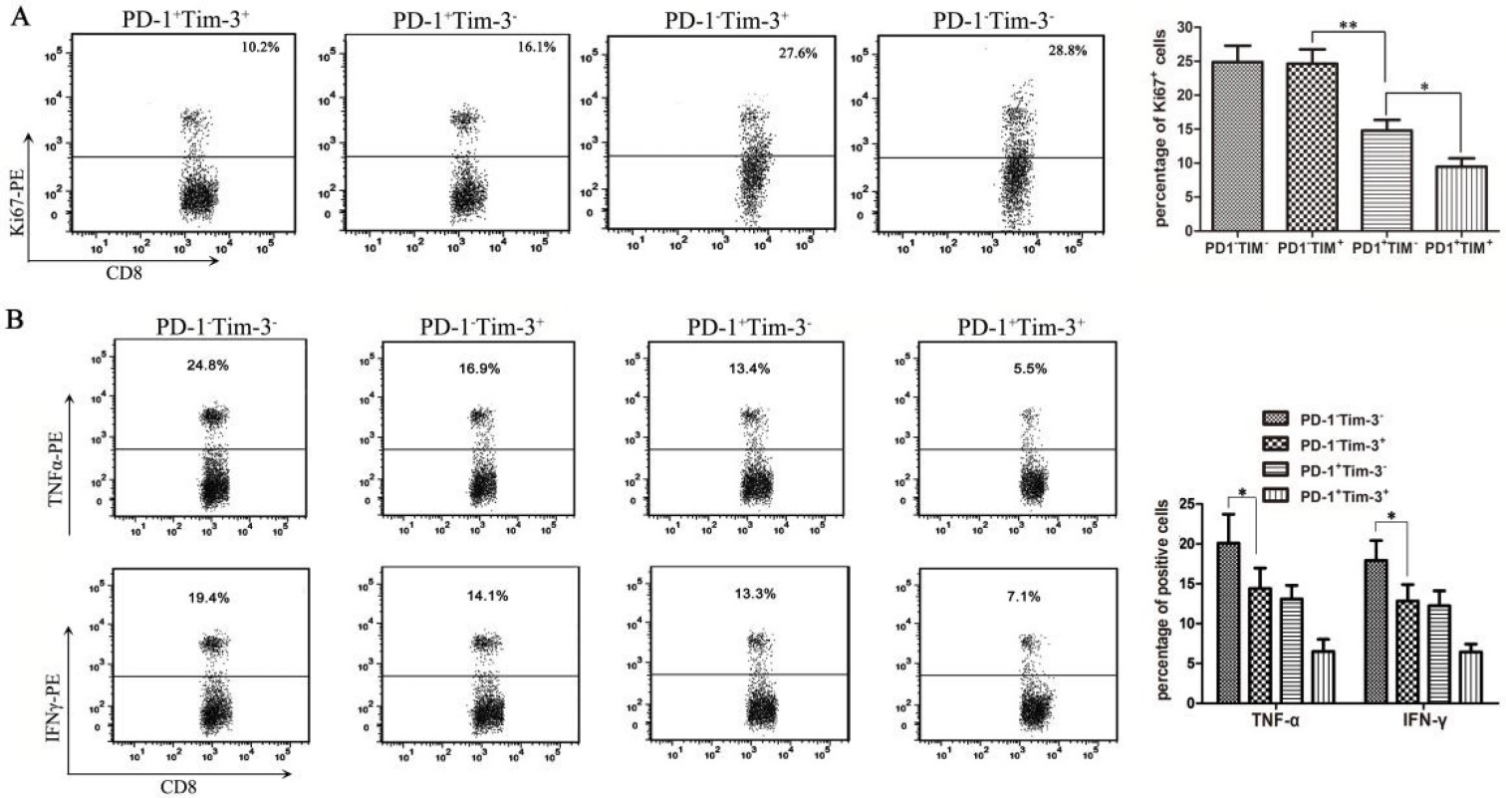
** Analysis of proliferation and cytokine secretion functions in CD8^+^ TILs from bladder cancer-bearing mice.** CD8^+^ TIL subsets were isolated from bladder cancer-bearing mice by a Flow Cell Sorter. (A) CD8^+^ TIL subsets were stained with an anti-Ki67 antibody, and the proliferation functions in each subset were detected by flow cytometry (^*^P<0.05). (B) After stimulation with anti-CD3/CD28 beads and protein transport inhibitor for six hours, CD8^+^ TIL subsets were stained with anti-IFNγ and anti-TNFα antibodies and the cytokine secretion functions in each subset were detected by flow cytometry (^*^P<0.05).

Many studies have shown that the inhibitory receptors of PD-1 and Tim-3 are upregulated by dysfunctional tumor antigen-specific CD8^+^ T cells 22. To further evaluate the functional exhaustion status of CD8^+^ TILs based on PD-1 and Tim-3 expression, in our series, CD8^+^ TIL subsets were first isolated from bladder cancer-bearing mice by a Flow Cell Sorter into the following subsets: PD-1^+^Tim-3^-^, PD-1^-^Tim-3^+^, PD-1^+^Tim-3^+^, and PD-1^-^Tim-3^-^. To analyze the proliferation function, these CD8^+^ TIL subsets were stained with an antibody against Ki67 after permeabilization. Then, Ki67 production in each CD8^+^ TIL subset was detected by flow cytometry. The results showed that the PD-1^+^Tim-3^+^ CD8^+^ TILs appeared to be less proliferative (Figure 5A). In contrast, the PD-1^-^Tim-3^+^ and PD-1^-^Tim-3^-^ CD8^+^ TIL subsets showed similar proliferation compared to the PD-1^+^Tim-3^-^ CD8^+^ TIL subset (Figure 5A). Moreover, we detected the capacity of cytokine secretion in different CD8^+^ TIL subsets after stimulation with anti-CD3/CD28 beads and protein transport inhibitor for 6 hours. PD-1^+^Tim-3^+^ and PD-1^-^Tim-3^-^ CD8^+^ TILs showed the least and most IFNγ and TNF-α production, respectively, among all CD8^+^ TIL subsets (Figure 5B). Thus, PD-1^+^Tim-3^+^ TILs were considered to be the most dysfunctional TIL subset, with low proliferation and cytokine secretion functions. Interestingly, PD-1^-^Tim-3^+^ TILs were highly proliferative but defective in cytokine production, so we could consider that PD-1 expression was closely associated with CD8^+^ TIL exhaustion, and Tim-3 expression was closely correlated with secretion function but not proliferation of CD8^+^ TILs.

### Sequential administration of anti-PD-1 and anti-Tim-3 improved the efficacy of SA-GM-CSF-anchored vaccine therapy

**Figure 6:**
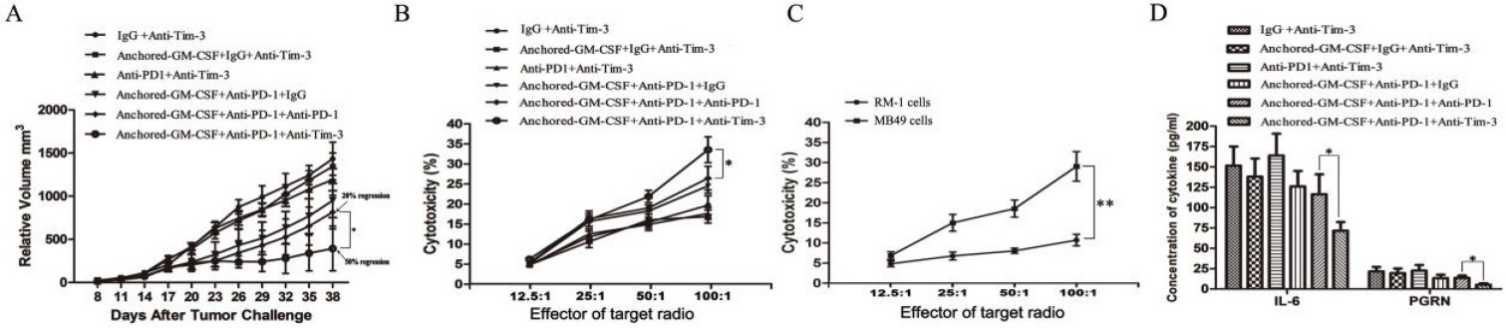
** Sequential administration of anti-PD-1 and anti-Tim-3 improved the efficacy of SA-GM-CSF-anchored vaccine therapy.** (A) All mice were injected subcutaneously with 1×10^6^ MB49 cells, and the tumor volume was recorded. Sequential administration of PD-1 and Tim-3 blockade with SA-GM-CSF-anchored vaccine treatment further inhibited the tumor growth compared with the other control groups (*P<0.05). (B) After the treatment was finished, splenocytes from each group were isolated and stimulated by IL-2. MB49 cells served as target cells. Supernatants were collected for a nonradioactive cytotoxicity assay (*P<0.05). (C) After the treatment was finished, splenocytes from mice treated with the sequential administration of PD-1 and Tim-3 blockade and the SA-GM-CSF-anchored vaccine were isolated and stimulated by IL-2. MB49 or RM-1 cells served as target cells. Supernatants were collected for a nonradioactive cytotoxicity assay (*P<0.05). (D) After the treatment was finished, peripheral blood was collected from each group and congealed and then the supernatants were harvested by centrifugation. The concentrations of IL-6 and progranulin (PGRN) were measured by ELISA (*P<0.05). All the experiments were replicated 3 times.

After subcutaneous tumors were established on day 8, SA-GM-CSF-anchored vaccine, anti-PD-1 and anti-Tim-3 treatments were given at certain time points (Figure 2). As shown in Figure 6A, sequential administration of anti-PD-1 and anti-Tim-3 combined with the SA-GM-CSF-anchored vaccine (triple therapy) significantly suppressed tumor growth, and tumor regression was noted in over 50% of the treated mice.

To investigate the antitumor immunity, the percent of cytotoxicity in each group was determined as the LDH activity of each well, and RM-1 prostate cancer cells were used to further confirm specific antitumor immunity (Figure 6B, C). Sequential administration of PD-1 and Tim-3 blockade in combination with SA-GM-CSF-anchored vaccine treatment effectively improved the cytotoxic activity of CTLs and established tumor-specific T cell immunity.

Some studies had found that checkpoint blockade can affect immune suppressive cytokine production in the TME 21,23. In this study of the MB49 model, we found that the concentrations of IL-6 and PGRN were significantly reduced with sequential treatment of anti-PD-1 and anti-Tim-3 (Figure 6D). IL-6 has been recognized as a pleiotropic cytokine with an obvious tumor-promoting effect 24. PGRN is a regulator of tumorigenesis because it stimulates cell proliferation, migration, invasion, angiogenesis, malignant transformation, resistance to anticancer drugs, and immune evasion 25. These results suggested that Tim-3 blockade might not only increase the infiltration of CD8^+^ TILs in the TME and enhance the cytotoxicity of specific antitumor CD8^+^ T lymphocytes but also decrease the levels of tumor-promoting cytokines.

## Discussion

Immunotherapy is an effective approach to enhance the antitumor immune response. The preexisting antitumor T cells in the TME are necessary for the efficacy of immunotherapy 26. Increasing effector T cell infiltration is closely correlated with longer survival 27. In our study, we found that SA-GM-CSF-anchored vaccine treatment alone could induce DC activation, enhance effector T cell infiltration into the TME and effectively improve the cytotoxic activity, but the vaccine was insufficient to inhibit tumor growth in established subcutaneous models of bladder cancer. Activated tumor-specific T cells usually produce inhibitory receptors, which can engage coinhibitory ligands on some tumor cells. This engagement induces negative regulatory pathways that limit normal immune responses, thus allowing the tumor to evade or dampen the immune response 28.

To confirm this phenomenon, we detected the expression of PD-1 on CD8^+^ TILs and PD-L1 in the TME in our MB49 model and found that this inhibitory receptor and ligand expression was upregulated in treatment with the SA-GM-CSF-anchored vaccine. Then, we used an anti-PD-1 antibody to prevent the binding of PD-L1 to PD-1 and found that the combination therapy with PD-1 blockade and the SA-GM-CSF-anchored vaccine synergistically induced antitumor immune responses, which greatly inhibited tumor growth, and even induced regression of established tumors. Moreover, to directly explore the associated adaptive immune resistance mechanism, in the *in vivo* experiment, we found that an anti-IFNγ antibody effectively inhibited PD-L1 expression in the TME. Because in our previous studies we confirmed that the SA-GM-CSF-anchored vaccine significantly increased the level of IFNγ 19, we considered that upregulated PD-L1 expression in the TME was dependent on IFNγ. Moreover, we also found that an anti-IFNγ antibody effectively abrogated the tumor regression. A previous study confirmed that sustained low-level expression of IFNγ could promote tumor development 29, so we could consider that IFNγ is a “double-edged sword” in antitumor efficacy in the MB49 model.

Although the combination therapy with PD-1 blockade and the SA-GM-CSF-anchored vaccine induced a better antitumor immune response than PD-1 blockade or the SA-GM-CSF-anchored vaccine alone, some of the mice still exhibited eventual tumor progression, the tumor regression rate was too low (20% regression). This finding was consistent with those of others who had suggested that a “checkpoint” blockade alone could not completely reverse the immune resistance because compensatory pathways were activated when a “checkpoint” was blocked 10,21. In this study, we found that Tim-3 expression was significantly upregulated in the anti-PD-1+SA-GM-CSF-anchored and anti-PD-1 groups, but not in the SA-GM-CSF-anchored group. This finding was consistent with recent research showing that Tim-3 positivity was significantly correlated with the duration of PD-1 blockade 21. This compensatory upregulation of Tim-3 expression may be a specific adaptive response to sustain the dysfunction of CD8^+^ TILs.

Several studies have shown that the differences in expression of inhibitory receptors (PD-1 and Tim-3) might result in different functions of tumor-specific CD8^+^ T cells 20,30. Therefore, it was necessary to evaluate the functions of T cells expressing different immune checkpoint receptors. We further studied CD8^+^ TIL subsets to provide a more precise phenotypic and functional profile of TILs. After *ex vivo* T cell receptor (TCR) stimulation of each sorted TIL subset, we found that PD-1^+^Tim-3^+^ CD8^+^ TILs were the most dysfunctional in terms of Th1 cytokine production and proliferation. Interestingly, PD-1^-^Tim-3^+^ CD8^+^ TILs were highly proliferative but defective in cytokine production. Because the characteristics of T cell exhaustion are failure to proliferate and exert effector functions in response to antigen stimulation 31, we considered that PD-1 expression was intensely associated with CD8^+^ TIL exhaustion and that Tim-3 expression was closely correlated with secretion but not proliferation of CD8^+^ TILs. Therefore, Tim-3 expression did not imply exhaustion of CD8^+^ TILs. This result was different from the traditional concept that Tim-3 expression was a biomarker of CD8^+^ TIL exhaustion 29,32.

Our studies found that Tim-3 expression on CD8^+^ TILs was upregulated via treatment with an anti-PD-1 antibody. In addition, Tim-3 expression decreased the secretory function of CD8^+^ TILs. Then, we combined SA-GM-CSF-anchored vaccine treatment with blockade of PD-1 and Tim-3 in an established subcutaneous bladder cancer model and found that this combination therapy significantly suppressed tumor growth, and tumor regression was increased in over 50% of the mice. The improvement of specific cytotoxic activity, together with a reduction in tumor-promoting cytokines, indicated that sequential administration of anti-PD-1 and anti-Tim-3 in treatment with the SA-GM-CSF-anchored vaccine induced a more robust antitumor immune response.

Anti-PD-1 immunotherapy is clinically effective in some bladder cancer patients. However, this promising immunotherapy still lacks a complete response despite the high frequency of PD-1^+^ effector T cells in the TME 10. The limited efficacy of PD-1 blockade to reinvigorate dysfunctional T cells could be partly explained by the presence of Tim-3 on a subset of the PD-1^+^ CD8^+^ TILs. PD-1^+^Tim-3^+^ CD8^+^ TILs may represent a negative prognostic biomarker of anti-PD-1 therapy unless the function of these cells can be restored with Tim-3 blockade. Our studies have demonstrated that dual blockade of PD-1 and Tim-3 induced a more robust antitumor immune response. Therefore, we conclude that the clinical response of anti-PD-1 immunotherapy should not only be evaluated in the context of PD-L1 expression in the TME but also in the frequency of PD-1^+^Tim-3^-^ CD8^+^ TILs, which may represent a positive prognostic biomarker of anti-PD-1 therapy.

Our study has some limitations that should be taken into account when interpreting our results. First, we focused on overcoming the vaccine-induced CD8^+^ TIL exhaustion/dysfunction in a model of bladder cancer (MB49 model). T cell exhaustion/dysfunction is double-edged and is promoted mainly by tumors. Therefore, the most accurate approach is to verify the results in multiple tumor cell lines. Second, in our study, we found the Tim-3 expression on CD8^+^ TILs was upregulated in response to PD-1 blockade. Tim-3 expression was detected following the PD-1 blockade, but the specific relationship between Tim-3 expression on CD8^+^ TILs and PD-1 blockade and the best time point of treatment via Tim-3 blockade in bladder cancer (MB49 model) needs to be further studied.

In summary, these results provide us with insight into understanding the dysfunction of CD8^+^ T cells induced by PD-1 and Tim-3 in the TME, as well as provide a rationale to combine SA-GM-CSF-anchored vaccine therapy with the blockade of PD-1 and/or Tim-3 to improve clinical outcome for bladder cancer patients.

## References

[1] Babjuk M, Burger M, Zigeuner R (2013). EAU guidelines on non-muscle-invasive urothelial carcinoma of the bladder: Update 2013. Eur Urol.

[2] Ferlay J, Soerjomataram II, Dikshit R, Eser S, Mathers C, Rebelo M (2015). Cancer incidence and mortality worldwide: sources, methods and major patterns in GLOBOCAN 2012. Int J Cancer.

[3] Singh NP, Yolcu ES, Taylor DD, Gercel-Taylor C, Metzinger DS, Dreisbach SK (2003). A novel approach to cancer immunotherapy: tumor cells decorated with CD80 generate effective antitumor immunity. Cancer Res.

[4] Hu Z, Tan W, Zhang L, Liang Z, Xu C, Su H (2010). A novel immunotherapy for superficial bladder cancer by intravesical immobilization of GM-CSF. J Cell Mol Med.

[5] Zhang X, Shi X, Li J, Hu Z, Zhou D, Gao J (2012). A novel therapeutic vaccine of mouse GM-CSF surface modified MB49 cells against metastatic bladder cancer. J Urol.

[6] Kulasinghe A, Perry C, Kenny L, Warkiani ME, Nelson C, Punyadeera C (2017). PD-L1 expressing circulating tumour cells in head and neck cancers. BMC Cancer.

[7] Liu J, Zhang S, Hu Y, Yang Z, Li J, Liu X (2016). Targeting PD-1 and Tim-3 Pathways to Reverse CD8 T-Cell Exhaustion and Enhance *Ex vivo* T-Cell Responses to Autologous Dendritic/Tumor Vaccines. J Immunother.

[8] Powles T, Eder JP, Fine GD, Braiteh FS, Loriot Y, Cruz C (2014). MPDL3280A (anti-PD-L1) treatment leads to clinical activity in metastatic bladder cancer. Nature.

[9] Kwon ED, Drake CG, Scher HI, Fizazi K, Bossi A, van den Eertwegh AJ (2014). Ipilimumab versus placebo after radiotherapy in patients with metastatic castration-resistant prostate cancer that had progressed after docetaxel chemotherapy (CA184-043): a multicentre, randomised, double-blind, phase 3 trial. Lancet Oncol.

[10] Bellmunt J, Powles T, Vogelzang NJ (2017). A review on the evolution of PD-1/PD-L1 immunotherapy for bladder cancer: The future is now. Cancer Treat Rev.

[11] Monney L, Sabatos CA, Gaglia JL, Ryu A, Waldner H, Chernova T (2002). Th1-specific cell surface protein Tim-3 regulates macrophage activation and severity of an autoimmune disease. Nature.

[12] Zhu C, Anderson AC, Schubart A, Xiong H, Imitola J, Khoury SJ (2005). The Tim-3 ligand galectin-9 negatively regulates T helper type 1 immunity. Nat Immunol.

[13] Piao YR, Jin ZH, Yuan KC, Jin XS (2014). Analysis of Tim-3 as a therapeutic target in prostate cancer. Tumour Biol.

[14] Cheng G, Li M, Wu J, Ji M, Fang C, Shi H (2015). Expression of Tim-3 in gastric cancer tissue and its relationship with prognosis. Int J Clin Exp Pathol.

[15] Yang M, Yu Q, Liu J, Fu W, Cao Y, Yu L (2015). T-cell immunoglobulin mucin-3 expression in bladder urothelial carcinoma: Clinicopathologic correlations and association with survival. J Surg Oncol.

[16] Fourcade J, Sun Z, Pagliano O, Chauvin JM, Sander C, Janjic B (2014). PD-1 and Tim-3 regulate the expansion of tumor antigen-specific CD8+ T cells induced by melanoma vaccines. Cancer Res.

[17] Zhang X, Shi X, Li J (2018). PD-1 Blockade Overcomes Adaptive Immune Resistance in Treatment with Anchored-GM-CSF Bladder Cancer Cells Vaccine. J Cancer.

[18] Fourcade J, Sun Z, Benallaoua M, Guillaume P, Luescher IF, Sander C (2010). Upregulation of Tim-3 and PD-1 expression is associated with tumor antigen specific CD8+ T cell dysfunction in melanoma patients. J Exp Med.

[19] Shi X, Zhang X, Li J, Zhao H, Mo L, Shi X (2017). PD-1/PD-L1 blockade enhances the efficacy of SA-GM-CSF surface-modified tumor vaccine in prostate cancer. Cancer Lett.

[20] Jie HB, Srivastava RM, Argiris A, Bauman JE, Kane LP, Ferris RL (2017). Increased PD-1+ and Tim-3+ TILs during cetuximab therapy inversely correlates with response in head and neck cancer patients. Cancer Immunol Res.

[21] Koyama S, Akbay EA, Li YY, Herter-Sprie GS, Buczkowski KA, Richards WG (2016). Adaptive resistance to therapeutic PD-1 blockade is associated with upregulation of alternative immune checkpoints. Nat Commun.

[22] Ngiow SF, von Scheidt B, Akiba H, Yagita H, Teng MW, Smyth MJ (2011). Anti-Tim3 antibody promotes T cell IFN-gamma-mediated antitumor immunity and suppresses established tumors. Cancer Res.

[23] Akbay EA, Koyama S, Carretero J, Altabef A, Tchaicha JH, Christensen CL (2013). Activation of the PD-1 pathway contributes to immune escape in EGFR-driven lung tumors. Cancer Discov.

[24] Knüpfer H, Preiss R (2007). Significance of interleukin-6 (IL-6) in breast cancer (review). Breast Cancer Res Treat.

[25] Arechavaleta-Velasco F, Perez-Juarez CE, Gerton GL, Diaz-Cueto L (2017). Progranulin and its biological effects in cancer. Med Oncol.

[26] Sweis RF, Spranger S, Bao R, Paner GP, Stadler WM, Steinberg G (2016). Molecular Drivers of the Non-T Cell-Inflamed Tumor Microenvironment in Urothelial Bladder Cancer. Cancer Immunol Res.

[27] Sharma P, Shen Y, Wen S, Yamada S, Jungbluth AA, Gnjatic S (2007). CD8 tumor-infiltrating lymphocytes are predictive of survival in muscle-invasive urothelial carcinoma. Proc Natl Acad Sci USA.

[28] Das M, Zhu C, Kuchroo VK (2017). Tim-3 and its role in regulating anti-tumor immunity. Immunol Rev.

[29] He YF, Wang XH, Zhang GM, Chen HT, Zhang H, Feng ZH (2005). Sustained low-level expression of interferon-gamma promotes tumor development: potential insights in tumor prevention and tumor immunotherapy. Cancer Immunol Immunother.

[30] Shayan G, Srivastava R, Li J, Schmitt N, Kane LP, Ferris RL (2016). Adaptive resistance to anti-PD1 therapy by Tim-3 upregulation is mediated by the PI3K-Akt pathway in Head and Neck Cancer. Oncoimmunology.

[31] Wherry EJ (2011). T cell exhaustion. Nature immunology.

[32] Wherry EJ, Kurachi M (2015). Molecular and cellular insights into T cell exhaustion. Nat Rev Immunol.

